# Molecular Analysis of Genetic Diversity and Structure of the Lablab (*Lablab purpureus* (L.) Sweet) Gene Pool Reveals Two Independent Routes of Domestication

**DOI:** 10.3390/plants12010057

**Published:** 2022-12-22

**Authors:** Alisa Kongjaimun, Yu Takahashi, Yosuke Yoshioka, Norihiko Tomooka, Rachsawan Mongkol, Prakit Somta

**Affiliations:** 1 Faculty of Animal Sciences and Agricultural Technology, Silpakorn University, Phetchaburi IT Campus, 1 Moo 3 Sampraya, Cha-am, Phetchaburi, Bangkok 10330, Thailand; 2 Genetic Resources Center, National Agriculture and Food Research Organization, Kannondai 2-1-2, Tsukuba 305-8602, Ibaraki, Japan; 3 Faculty of Life and Environmental Sciences, University of Tsukuba, 1-1-1 Tennodai, Tsukuba 305-8572, Ibaraki, Japan; 4 Department of Agronomy, Faculty of Agriculture at Kamphaeng Saen, Kasetsart University, Kamphaeng Saen Campus, 1 Moo 6 Kamphaeng Saen, Nakhon Pathom 73140, Thailand

**Keywords:** hyacinth bean, diversity, domestication, SSR, chloroplast

## Abstract

In this study, genetic diversity and structure of 474 cultivated and 19 wild lablab (*Lablab purpureus*) accessions. were determined using 15 nuclear and 6 chloroplast SSR markers. The overall gene diversity was relatively low (0.3441). Gene diversity in the wild accessions (0.6059) was about two-folds greater than that in the cultivated accessions. In the wild accessions, gene diversity was greatest in the southern Africa, followed by East Africa. In the cultivated accessions, gene diversity was highest in the eastern Africa. The results suggested that South Africa is the center of origin and East Africa is the center of domestication of lablab. Different cluster analyses showed that 2-seeded-pod cultivated accessions (ssp. *uncinatus*) were clustered with wild accessions and that 4–(6)-seeded-pod cultivated accessions (ssp. *purpureus* and *bengalensis*) were intermingled. UPGMA tree suggested that ssp. *purpureus* and *bengalensis* were domesticated from 4-seeded-pod wild accessions of southern Africa. Haplotype network analysis based on nuclear SSRs revealed two domestication routes; the ssp. *uncinatus* is domesticated from 2-seeded-pod wild lablab (wild spp. *uncinatus*) from East Africa (Ethiopia), while the ssp. *purpureus* and *bengalensis* are domesticated from 4-seeded-pod wild lablab from Central Africa (Rwanda). These results are useful for understanding domestication and revising classification of lablab.

## 1. Introduction

Lablab or hyacinth bean (*Lablab purpureus* (L.) Sweet) is one of the most ancient and important tropical legume crops of the world. This legume is widely cultivated throughout tropical and sub-tropical regions [1]. In general, cultivated and wild lablab plants are bushy, trailing or twining herbaceous with annual or biennial or perennial and indeterminate growth habits, although some improved lablab cultivars are short and non-bushy with annual and determinate growth habit. Lablab is mainly grown as field and vegetable crops by small-farm holders in Asia and Africa for human food in which young leaves, seeds and pods, and mature seeds are edible [2]. Dry seeds of lablab contain high protein content of about 25% of proteins and 60% of carbohydrates [3] and are rich in essential amino acids such as lysine and leucine [4,5]. Although dry seeds of lablab contain low lipid content of about only 1.2% [6], the lipids contain essential fatty acids, including linoleic acid and alpha-linolenic acid [5]. Moreover, the seeds contain several micronutrients and minerals [5,7]. While, lablab leaves contain 15 to 40% of proteins [8]. Thus, lablab seeds are a good source of proteins and carbohydrates, while young lablab pods and leaves are good sources of vitamins and minerals for people. In some countries such as India and Australia, the crop is also grown as forage crop, cover crop and green manure crop [8,9,10]. In addition, it is often grown as a weed suppressor and a soil erosion retardant [2,11]. Lablab can grow in a wide range of climate conditions and soil types due to its tolerance to drought, salinity and high temperature [12,13,14]. The crop resists and survives under drought condition by developing deep tap root up to 2 m or deeper and tuber-like root which can regrow when suitable environment arrives [14,15]. Due to its high nutrition, multi-propose uses and drought tolerance, lablab can be one of legume crops suitable for tropical regions to mitigate effects climate change. 

Despite lablab is a versatile crop, the potential of this crop has not been fully utilized and there is a limited number of reports on genetic diversity of hyacinth bean. Most of the lablab cultivars grown in the world are landraces or pure lines selected from landraces, except in India, Bangladesh, China, Australia, USA and some European countries where improved cultivars are developed by hybridization and selection [2,15,16]. There are not many breeding programs for lablab and most of them are small and local programs conducted in developing and underdeveloped countries. Lablab is the only species of the genus *Lablab* and three subspecies (ssp.), *uncinatus*, *purpureus* and *bengalensis*, have been described and accepted for this species [17]. The *uncinatus* has two forms, wild and cultivated, while the the *purpureus* and *bengalensis* are cultivated form. These three ssp. generally show similar phenotypic traits. The key traits used to classify and differentiate them are pod shape, pod size, and seeds per pod. Pods of the spp. *uncinatus* and *purpureus* are crescent-like to more or less straight and oblong, or also dorsally straight and ventrally deeply curving while suddenly near the top returning towards the slender beak, laterally compressed, and bulging over the seeds [18]. These two subspecies are differentiated by pod size and seeds per pod; the former has pods of about 4 cm in length and 1.5 cm in width, while the latter has larger pods than the *uncinatus*, up to 10 cm in length and about 4 cm in width [17]. In contrast, the subspecies *bengalensis* has longer pods than the *purpureus*, narrowly oblong or linear-oblong, up to 14 cm in length and about 1-2.5 cm in width [17]. Nonetheless, wild form of the *uncinatus* is believed to be the progenitor of all the cultivated forms [10]. Lablab is an ancient legume crop of the world. The oldest archaeo-botanical finds of lablab is found in India and is dated 2000 to 1700 BC [19]. 

Lablab is believed to be originated in Africa where its wild formed is widely found in natural habitats [17]. There are not many reports genetic diversity study of the lablab, especially at the molecular level [9,10,11,12,15,20,21,22,23,24,25,26]. However, extent of gene pool diversity and population structure of this legume is still poorly understood as nearly all of this use small number of germplasms from Africa or Southeast Asia or India (<150 accessions) and low-informative DNA markers. Nonetheless, population structure analysis in a set of 91 lablab accessions (4, 7 and 80 were subsp. *uncinatus*, *bengalensis* and *purpureus*, respectively) from various origins using 6 simple sequence repeat (SSR) markers revealed that (i) only some accessions of the ssp. *purpureus* from Ethiopia, Malawi, Kenya and Zimbabwe were most closely the wild lablab accessions (spp. *uncinatus*), (ii) accessions of the ssp. *purpureus* and *bengalensis* are not distinctly different, and (iii) accessions of the ssp. *purpureus* were the most diverse among the cultivated germplasm [12]. In the same study, the analysis based on a chloroplast DNA sequence showed 2 haplotypes, A and B, in the lablab germplasms [12]. The haplotype A is unique to the wild accessions (ssp. *uncinatus*) and four accessions of ssp. *purpureus* from Africa, whereas the haplotype B is found in all forms and origins of cultivated lablab [12]. These results indicate that the lablab is probably domesticated in East Africa. However, in that study the number of wild forms was very small (6 accessions), the number of markers used was very limited (6 markers) and wild form with 4-seeded pods were not included. So, the results and conclusions obtained from that study may not precisely reflect the gene pool diversity, population structure of the lablab.

In this study, we investigated genetic diversity and population structure in a large collection of lablab germplasm originating from Africa, America, Asia, Europe and Oceania using SSR markers developed from nuclear DNA of hyacinth bean, azuki bean and mungbean, and chloroplast DNA from cowpea. We also developed a core collection of the lablab. 

## 2. Results

### 2.1. Morphological Variations in Lablab

In this study, 493 accessions of lablab were grown and evaluated for morphologocal variation. Variations in 14 morphological traits relating to stem, leaf, flower, pod and seeds are summarized in Table 1 (see also Supplementary Table S1). Both cultivated and wild accessions showed the same variation in stem color and dry pod color. There was no variation in leaf color in the wild accessions; all the accessions showed green leaves. However, the cultivated accessions showed purple and green leaves. The wild and cultivated accessions expressed different variations in flower colors. The wild accessions expressed purple flower, while the cultivated accessions expressed purple and white flowers. There was no variation in young pod color in wild accessions; all of them had green pods. On the contrary, the cultivated accessions showed green and purple pods. The cultivated accessions were statistically significant difference from the wild accessions in all the quantitative traits measured (Table 1). Compared to the wild accessions, cultivated accessions were larger in size of mature pods and seeds. The cultivated accessions had more seeds per pod than the wild accessions. 

### 2.2. Nuclear SSR Variation and Genetic Diversity of Lablab

Of the 27 nuclear SSR markers used to screen for polymorphism in the six lablab accessions, 15 were able to amplify the DNA and showed polymorphism. When the polymorphic markers were used to analyze the 493 lablab accessions, they detected 131 alleles in total (Table 2). The number of alleles detected per marker was between 2 (Hbp_012) and 19 (KTD245) with an average of 8.73. The polymorphism information content (PIC) values of these markers varied from 0.0083 (Hbp_012) and 0.6587 (c17963_g1_i1) with an average of 0.3167 (Table 2).

The overall observed heterozygosity (*H*_O_) was 0.0364. The *H*_O_ value in wild accessions (0.1417) was higher than that in the cultivated accessions (0.0325). In the cultivated accessions, *H*_O_ value was highest in accessions from Europe (0.0433) and lowest in the accessions from America. However, in the subregion level, the *H*_O_ value was highest in accessions from southern Africa (0.0519), followed by East Asia (0.0417) and lowest in the accessions from America (Table 3). The overall gene diversity (*H*_E_) was relatively low, being 0.3441. The *H*_E_ in the wild lablab (0.6059) was about two-folds higher than that in the cultivated lablab (0.3139). Among the cultivated accessions, the *H*_E_ was highest in the African accessions (0.3393), followed by Asian (0.3018), Australian (0.2426), European (0.2197), and American accessions (0.1869). However, the *H*_E_ value of the African accession and that of the Asian accessions were only slightly different (Table 2 and Table 3). In the Africa, the *H*_E_ was greatest in the East African accessions (0.3565), but not much different from that in the South African accessions (0.3158). In Asia, the *H*_E_ was highest in the South Asian accessions (0.3175), albeit only marginally different from that in the East (0.2467) and the Southeast Asian accessions (0.2370). 

*N*_A_, *H*_O_, and *H*_E_ of the different spp./types of the cultivated and wild lablabs were compared and are presented in Table 4. In the cultivated accessions, the *N*_A_ was highest in *purpureus* (6.60), followed by *bengalensis* (2.53) and *uncinatus* (1.47). The *H*_O_ of *uncinatus* (0.0689) was two-folds higher than that of *purpureus* (0.0332) and *bengalensis* (0.0306). Nonetheless, the *H*_E_ of *purpureus* (0.2971) was slightly higher than that of *bengalensis* (0.2584), but was more than two-folds higher than that of *uncinatus* (0.1222). In the wild accessions, the 4-seeded-pod accessions possessed higher *N*_A_ and *H*_E_, but lower *H*_O_ than the 2-seeded-pod accessions. 

### 2.3. Population Structure Analysis

**Figure 1:**
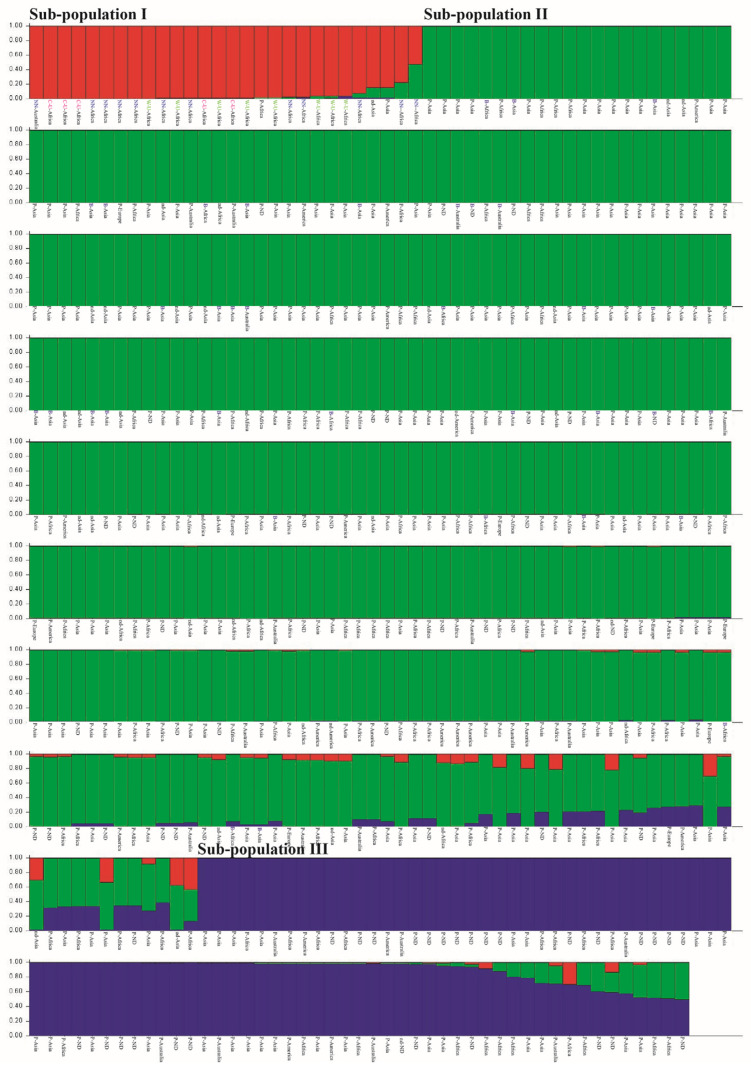
Population structure of the 493 lablab accessions determined by STRUCTURE analysis based on 15 nuclear SSR markers. Each bar represents one individual. B = spp. *bengalensis*, P = spp. *purpureus*, C-U = cultivated spp. *uncinatus*, W-U = wild spp. *uncinatus*, NN = wild ssp. *nomen nominandum*, and ND = not determined.

Bayesian clustering of the 493 lablab accessions was performed using STRUCTURE software. Based on Evanno’s *ad hoc* Δ*K* method [27], there were three sub-populations among the 493 accessions; subpopulations I, II and III (Figure 1). Sub-population I comprised 26 accessions; 22, 2, 1, and 1 accessions were from Africa, Asia, Australia and unknown, respectively. All the wild accessions of subspecies together with all of cultivated subspecies *uncinatus* and two cultivated of subspecies *purpureus* belonged to this sub-population. Sub-population II was the largest subpopulation having 382 cultivated accessions originating from Africa, America, Asia, Europe and Australia. All the 33 accessions of the subsp. *bengalensis* were in this sub-population, while rest of the accessions in this sub-population were the subsp. *purpureus*. Sub-population III comprised 85 accessions of which all of them were the subsp. *purpureus* originating from Africa, America, Asia and Australia.

### 2.4. UPGMA Analysis and Neighbor-Joining Analysis 

**Figure 2:**
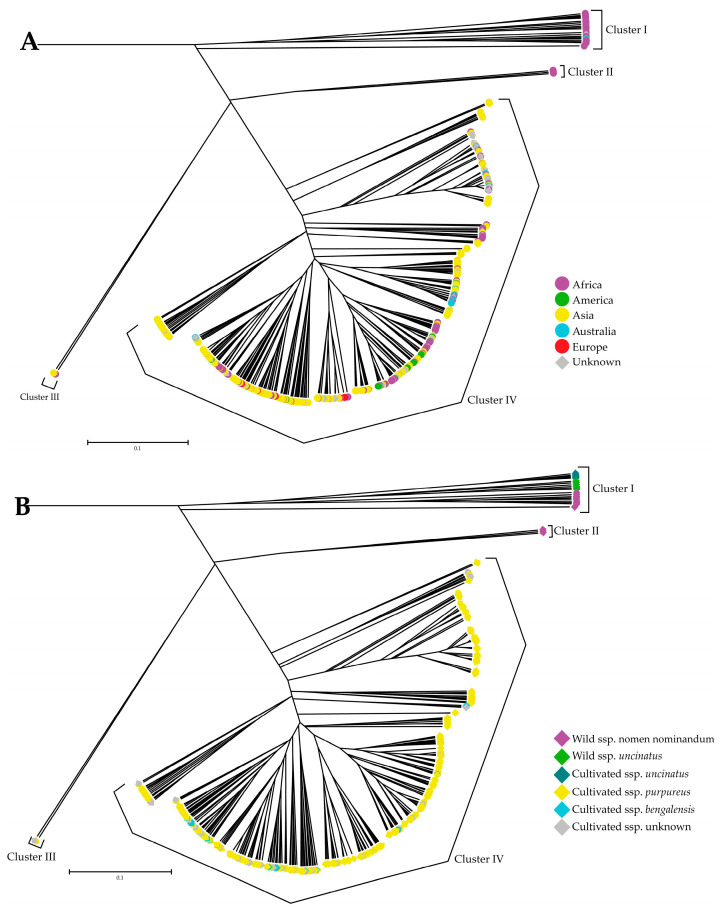
UPGMA tree of 493 lablab accessions based on *D*_A_ genetic distances [28] The distance was calculated from 15 nuclear SSR markers. (**A**) The accessions are presented based on their geographical origins. (**B**) The accessions are presented based on taxonomical classification.

**Figure 3:**
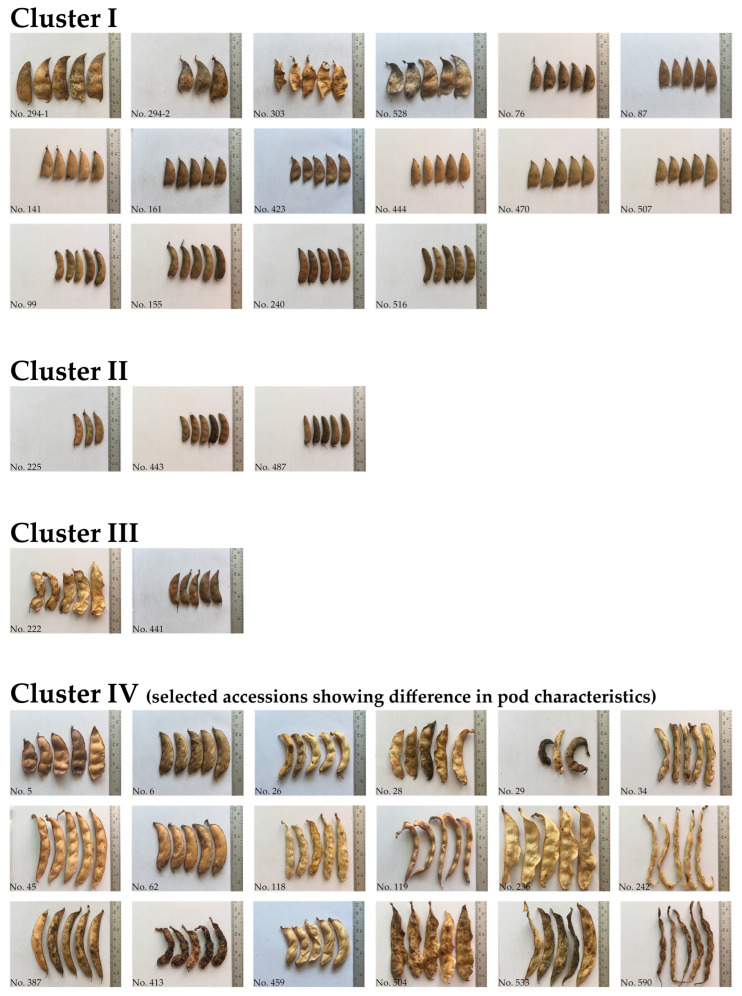
Pod characteristics of some lablab accessions in each genetic cluster determined by UPGMA cluster analysis.

**Figure 4:**
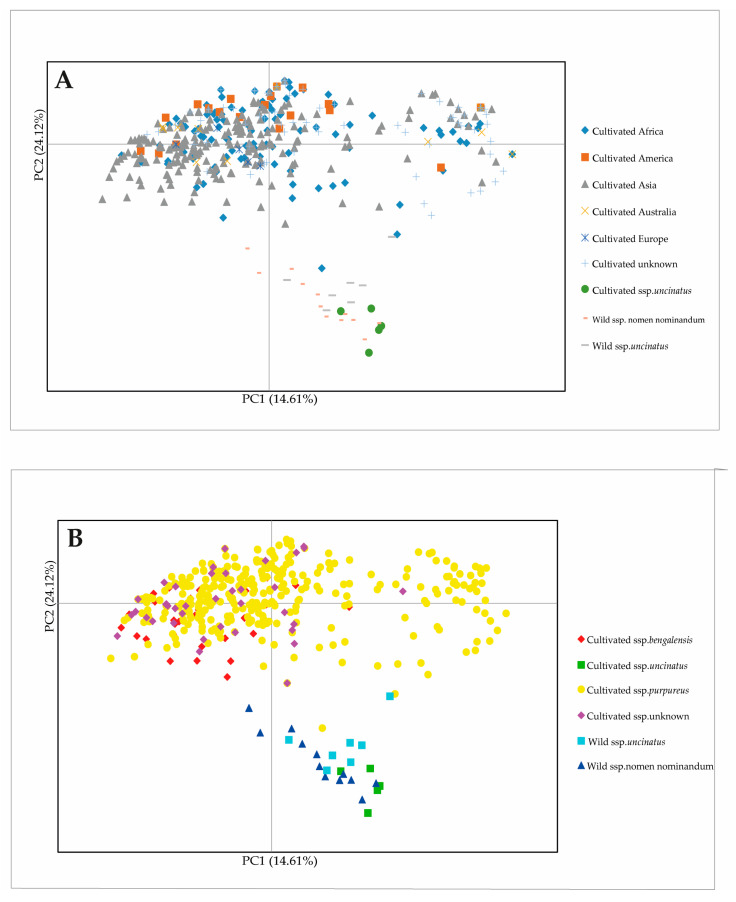
Scatter diagrams of 493 lablab accessions based on the first and second axes of principal coordinate analysis (PC1 and PC2). (**A**) The accessions are presented based on cultivation status and geographical origins. (**B**) The accessions are presented based on taxonomical classification.

Phylogenetic trees of the 493 lablab accessions were reconstructed based on *D*_A_ by the unweighted pair-cluster method using arithmetic averages (UPGMA) and neighbor-joining (NJ) methods. We found that although the two methods revealed different number of clusters, 2 for UPGMA (Figure 2) and 4 for NJ (Figure S1), the two methods provided similar patterns of germplasm clustering. However, we described the results of from the UPGMA analysis. The UPGMA tree revealed four clusters (I, II, III and IV) of the accessions (Figure 2A and Figure 3). In general, the cultivated accessions were clearly separated from the wild accessions. Nearly all of the cultivated accessions from Africa, America, Asia, Europe, and Australia were grouped together in a majority cluster (cluster IV). Accessions from different regions were intermingled. The wild accessions were separated into two clusters I and II. All of the cultivated and wild accessions (2-seeded-pod wild accessions) of the spp. *uncinatus* together with 8 of the 4-seeded-pod wild accessions were grouped into the cluster I. Four 4-seeded-pod wild accessions were grouped into the Cluster II. The cluster III was the smallest cluster containing only three cultivated accessions, one from Africa (No. 441) and two from India (No. 145 and No. 222). The No. 441 showed quite short pod with 3 seeds per pod, while the No. 145 and No. 222 showed long pod with 4 seeds per pod (Figure 2A and Figure 3). The UPGMA tree also demonstrated that the spp. *uncinatus* and the wild lablab were distinctly separated from the spp. *purpureus* and *bengalensis* (Figure 2B). The spp. *purpureus* and *bengalensis* were grouped together and not clearly separated in the cluster IV (Figure 2B). Nonetheless, in all cases, the bootstrap value at each node was low (<50). 

### 2.5. Principal Coordinate Analysis 

PCoA analysis based on *D*_A_ revealed that the first three PCs together accounted for 70.90% of the total variation. PC1, PC2 and PC3 explained 14.61, 24.12 and 32.17% of the total variation, respectively. A scatter plot of the 493 lablab accessions based on PC1 and PC2 showed that, in general, the cultivated accessions of the ssp. *uncinatus* and wild accessions were distributed close together and were clearly separated from accessions of the ssp. *purpureus* and *bangalensis*. Cultivated accessions of the ssp. *purpureus* and *bangalensis* were distributed together with no geographical pattern (Figure 4). 

### 2.6. Chloroplast SSR Variation and Haplotype Diversity of Lablab

**Figure 5:**
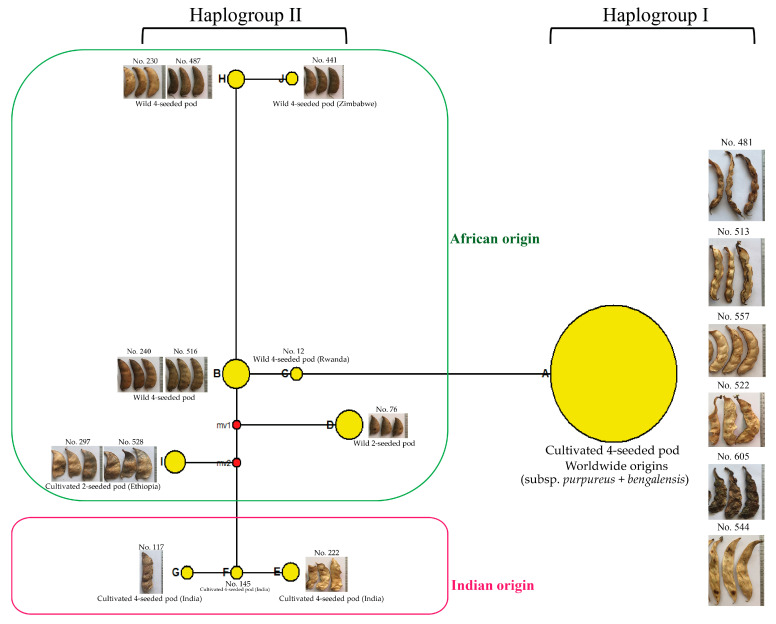
Scheme depicting haplotype network of 460 lablab accessions. Branch lengths is proportional to the number of mutational steps in 10 chloroplast haplotypes. Size of pie chart is proportional to the haplotype frequency. Mv1 and mv2 represent mean median vector.

Among 12 chloroplast SSR markers screened for polymorphism, six showed polymorphisms. Analysis of the six markers in all the 493 lablab accessions revealed 25 alleles in total with the *N*_A_ ranging from 3 to 5 and an average of 4.17 and the *H*_E_ varying between 0.0371(VgcpSSR14) to 0.1105 (VgcpSSR05) with an average of 0.948 (Table 5). Based on the chloroplast alleles detected by these SSRs, 10 haplotypes, designated A to J, were identified from the 493 lablab accessions. All the cultivated accessions with 4-6 seeds per pod except three accessions (No. 117, 145 and 222) belonged to haplotype A (Figure 5). The accessions No. 222, 145 and 117 were all from India and belonged to different haplotypes, E, F, and G, respectively. All the cultivated accessions with 2 seeds per pods, all from Africa, belonged to haplotype I. The wild accessions were classified into four haplotypes, B, C, D, H and J. The accessions in the haplotype D had 2-seeded pods, while the accessions in the haplotypes B, C, H and J had 4 seeds per pod However, it is noteworthy that haplotypes of 63 accessions including wild and cultivated types were not determined due to missing data on some chloroplast SSR markers.

Haplotypic data of 430 lablab accessions (63 accessed were excluded due to missing in some chloroplast markers) were used for Median–joining network analysis. The analysis showed that all the 10 haplotypes were clustered into 2 haplogroups (I and II). The haplogroup I was consisted of only haplotype A, which was the largest haplogroup. Accessions in this haplogroup were all cultivated accessions that originated from Africa, America, Asia, Europe, and Australia. The haplogroup II was consisted of haplotypes B to J. All the wild accessions (haplotypes B, C, D, H, I and J) and cultivated accessions No. 222, 145 and 117 from India (haplotypes E, F and G) were in this haplogroup (Figure 5). 

### 2.7. Core Collection Development of Lablab

Based on allelic data of 16 nuclear SSR markers in the 493 lablab accessions, a core collection of 47 accessions comprising 33 cultivated and 14 wild accessions were developed (Supplementary Table S1). The core collection had 131 alleles in total, gene diversity of 0.5744, and observed heterozygosity of 0.0812 (Table 6). Among the cultivated accession, 8, 2, 11, 1, and 9 were from Africa, America, Asia, Europe, Australia and unknown origin. Among the wild accessions, 12, 1, and 1 originated from Africa, and Australia and unknown origin. The core collection contained all the three known subspecies (*uncinatus* (9 accessions), *purpureus* (31 accessions), *bengalensis* (1 accession) and unknown subspecies (6 accessions of 4-seeded-pod wild).

## 3. Discussion

All previous molecular genetic diversity analyses in lablab were conducted using limited number of accessions (<150 accessions) from Africa or Southeast Asia or India with dominant molecular markers (AFLPs and RAPDs) [20,21,22,23] except for Zhang et al. [24] and Robotham and Chapman [12] that used codominant marker (SSRs). Our study was the largest assessment of genetic diversity conducted in lablab germplasm including 474 cultivated and 19 wild accessions (493 lablab accessions in the total) by using 15 nuclear and 6 chloroplast SSR markers (Table 2 and Table 5). 

### Center of Origins, Diversity and Domestication of Lablab

In this study SSR analysis showed that cultivated and wild lablab germplasms from Africa possessed the highest gene diversity (Table 3), suggesting that Africa is the center of origin and diversity of the lablab. This is in line with previous results obtained by morphological observation [17] and molecular marker analysis [10,12]. However, the gene diversity in Africa was only slightly different from that in Asia (Table 3). This suggested that Asia is a second center diversity of lablab. In our study, the gene diversity in the cultivated accessions was highest in East Africa, followed by that in South Asia, and South Africa (Table 3), while the gene diversity in the wild accessions was greatest in the South Africa, followed by that in the East Africa. These results supported the opinions of Verdcourt [17], Maass et al. [10] and Maass [29] that eastern and southern Africa are the center of origin of the lablab, and the results reported by Robotham and Chapman [12] that eastern Africa is the center of origin of lablab. Our results also suggested that South Asia is a second center of diversity of lablab. The haplotype network further suggested that the 2-seeded pods wild lablab (wild ssp. *uncinatus*) from the Ethiopia (East Africa) is the ancestral or founding haplotype (Figure 5; see also Supplementary Table S1), and hence the center of origin of the lablab. Notably, haplotypes of several wild lablab accessions with 2- and 4-seeded-pod types could not be determined. 

In a comprehensive analysis, Maass et al. [30] revisited previous results from diversity studies on lablab and integrated phenotypic data (pod- and seed-related traits) to the germplasm used in those studies, they proposed that the crop may experience two domestication events; one involved the 2-seeded pods and another one involved 4-seeded pods, and that Ethiopia is the most probable candidate area of lablab domestication because the certain accessions from Ethiopia are closely related with 2-seeded-pod wild lablab. A similar finding was observed in our study; UPGMA tree based on nuclear SSR markers clearly showed that the Ethiopian cultivated accessions with 2-seed pods clustered with the wild accessions (both 2- and 4-seeded pod types) (Figure 2 and Figure 3; see also Supplementary Table S1). In the domestication events proposed by Maass et al. [30], the cultivated lablabs with 4-seeded pods (ssp. *pupureous* and *bengalensis*) are domesticated from a (taxonomical uncertain) wild lablab with 4-seeded pods. In our study, the UPGMA clearly showed that a group of four wild accessions with 4-seeded pods from the southern Africa (two each from South Africa and Zimbabwe) were distinct from the other wild accessions and were the most closely related with the cultivated accessions with 4-seeded pods (Figure 2; see also Supplementary Table S1). These suggested that the ssp. *pupureous* and *bengalensis* are domesticated from the 4-seeded-pod wild lablab from southern Africa, probably in South Africa and Zimbabwe. The haplotype network based on the chloroplast SSR markers (Figure 5; see also Supplementary Table S1) also supported that the domestication of the ssp. *pupureous* and *bengalensis* from the 4-seeded-pod wild type (haplotype C). Nonetheless, the network suggested that the domestication of the 4-seeded-pod lablab took place in the Central Africa (Rwanda) and that the 2-seeded-pod wild lablab (wild ssp. *uncinatus*) from the East Africa is the ancestral or founding haplotype. So, the origin of domestication of 4-seeded pod lablab (ssp. *pupureus* and *bengalensis*) is still unclear. One of the problems in studying evolution of lablab is taxonomical classification of subspecies [30] where wild variants with different number of seeds per pods are all lumped into the ssp. *uncinatus* (2-seeded-pod type) [17], although 4-seeded-pod wild lablab had been proposed as ssp. *crenatifructus* [30,31]. In addition, the cultivated lablabs with 4(-6)-seeded pods are classified into two ssp. *pupureus* and *bengalensis* based mainly on their pod characteristics. Nonetheless, our results clearly showed that accessions of the ssp. *pupureous* and *bengalensis* are not genetically different (Figure 1, Figure 2, Figure 3 and Figure 4). These results are in line with previous studies [10,12,15,30]. We, therefore, agreed with Maass et al. [30] who noted that taxonomy of the lablab should be revised. In addition, we proposed that the “cultivar group” concept for the lablab [18,31] should be re-considered in the taxonomic revision of the lablab. However, additional analysis of chloroplast and/or mitochondrial genome using a large and comprehensive set of lablab germplasm should be carried out to provide a better insight into the domestication. 

Three of the cultivated lablab accessions having 4-seeded pods, viz. No. 222 and 145 from India and No. 441 from Africa were distinctly separated from the other cultivated accessions with 4-seeded pods and showed the closest genetic relationship with a group of wild accessions 4-seeded pods (Figure 2). In the population structure analysis, these accessions were clustered with wild accessions (Figure 1). In the haplotype analysis, No. 145 and 222 possessed different haplotypes from all the other accessions (Figure 5) and appeared to be closely related with cultivated accession with 2-seeded pods (ssp. *uncinatus*). Based on the passport data, the No. 222 and 145 were collected from wild habitats. Hence, the accessions No. 145, 222 and 441 are likely to be primitive lablab cultivars that escaped from cultivation, albeit the evolution of these accessions are still unclear. These accessions are value germplasm for future use in lablab breeding.

In this study, we developed a core collection of 47 lablab accessions. The core collection represented 9.53% of the original collections (493 accession) used in the study. This is nearly the same with the proportion for core collection (10%) proposed by Frankel and Brown [32]. The core collection contained the same number of alleles found in the original collection, but a much higher gene diversity (Table 6). This core collection comprised both wild and cultivated accessions, and thus it will be useful for evaluating traits of importance such as resistance to insects and diseases, plant types, and yield. 

The present study is the first large-scale genome level analysis of the lablab gene pool. Although the lablab germplasm collection analyzed is poorly represented in germplasm from some areas, particularly wild lablab from West and Central Africa, the relationships among components of the lablab gene pool and two independent routes of domestication of lablab have been revealed. The results from this study should assist breeders in selecting lablab germplasm for evaluation and use in breeding programs and plant taxonomists in classifying the intraspecies of lablab. 

## 4. Materials and Methods

### 4.1. Lablab Germplasm and DNA Extraction

In total, 493 (474 cultivated and 19 wild) accessions of lablab originating from various origins including Africa (137 accessions), America (22 accessions), Asia (237 accessions), Europe (5 accessions), Australia (16 accessions), and unknown origin (76 accessions) were used in this study (Supplementary Table S1). Among these accessions, 5, 397, 33, and 39 were cultivated accessions of the spp. *uncinatus*, *purpureus*, *bengalensis*, and unknown spp., while 7 and 12 accessions were wild accessions with 2-seeded pods (wild spp. *uncinatus*) and 4-seeded pods (wild ssp. *nomen nominandum* (as proposed by Maass et al. [30])). All the accessions were grown in an experimental field of Faculty of Animal Sciences and Agricultural Technology, Silpakorn University, Phetchaburi IT Campus, Phetchaburi, Thailand during August 2018 to August 2019.

Young leaves from a single plant of each accession were collected and extracted for total genomic DNA. The DNA extraction was carried out using a CTAB method [33]. DNA concentration was adjusted with a known concentration of lambda DNA using 1.5% agarose gel electrophoresis.

### 4.2. Characterization of Phenotypic Traits

Four-teen traits relating to stem, leaf, flower, pod, and seeds including stem color, leaf color, flower color, days to first flowering, fresh pod length (cm), fresh pod width (cm), dry pod length (cm), dry pod width (cm), fresh pod color, dry pod color, deed length (mm), seed width (mm), deed thickness (mm), and number of seeds per pod (count) (Table 7) were determined. 

### 4.3. Nuclear and Chloroplast SSR Markers Analysis

A total of 27 nuclear SSR markers were used to screen for polymorphism in six lablab accessions (No.28, 76, 119, 130, 528 and 606) originating from different geographic regions. Among these markers, 22, 5, and 1 were from lablab [12,24,34], azuki bean [35,36], and mungbean [37], respectively (Supplementary Table S2). In addition, they previously showed polymorphism in a collection of lablab germplasm of Thailand [15]. A polymerase chain reaction (PCR) mixture was prepared in a total volume of 10 µL containing 2.0 µL of template DNA, 5 µL of 2× QIAGEN Multiplex PCR Master Mix (Qiagen, Germany), 1.0 µL of Q-solution, 0.01 µL of 100 uM primers mix. The 5’-end of the reverse primer was fluorescent labeled with one of the three following fluorescent dyes: Fam Hex, and NED (Applied Biosystems, CA, USA). PCR reactions were performed in a GeneAmp PCR System 9700 (Applied Biosystems, CA, USA). The PCR thermal cycling was programmed as follows: 95 °C for 15 min followed by 40 cycles of 94 °C for 30 s, 60 °C for 90 s, 72 °C for 60 s, and a final extension at 72 °C for 30 min. After amplification, 1 µL of PCR product was mixed with 10 µL of Hi-Di formamide and 0.125 µL of ROX™ Size Standard (Applied Biosystems, CA, USA) and run on an ABI Prism 3100 or 3130xl Genetic Analyzer (Applied Biosystems, CA, USA). Allele size for the highest stutter peak with the height ranging between 500 and 10,000 relative fluorescence units (RFU) were recorded and used to create bins for automatic assignment of genotypes. The genotyping was conducted by the GeneMapper 3.0 software (Applied Biosystems, CA, USA) with default settings. After marker screening, two or four differentially labeled primers were mixed into a single PCR reaction mixture and amplified. Fluorescent signal strengths of each amplified fragment were leveled by increasing nonfluorescent labeled primer pairs while reducing the labeled primers. Such multiplex sets were used to genotype all the lablab accessions.

To analyze haplotypes of the lablab germplasm, 12 chloroplast SSR markers developed from *Vigna unguiculata* reported by Pan et al. [38] were used to screen for polymorphism in 24 lablab accessions originating from different countries and showing different phenotypic traits (Supplementary Table S2). Chloroplast SSR marker analysis were the same for the nuclear SSR marker as described above.

### 4.4. Genetic Data Analysis

Allelic data from the nuclear SSR markers were used to calculate number of alleles, the major allele frequency, observed heterozygosity (*H*_O_) and expected heterozygosity (gene diversity; *H*_E_) in the 493 lablab accessions using PowerMarker 3.25 software [39]. Polymorphic information content (PIC) which measure discriminatory power of DNA marker [40] was calculated for each nuclear SSR marker using the PowerMarker. 

Population structure of the 493 lablab accessions was determined from nuclear SSR allele data by STRUCTURE analysis [41] using STRUCTURE 2.3.4 software [41]. Initially, a 20-simulation run was carried out with number of assumed populations (*K*) ranging from 1 to 10 and burn-in period of 10,000 and 50,000 replicates of Bayesian Markov Chain Monte Carlo (MCMC) algorithm. The outputs from the simulation run were used to estimate the number of *K* using the ad-hoc Δ*K* method [27]. Subsequently, a run with optimum *K*, burn-in period of 100,000 and 500,000 replicates of the MCMC algorithm was performed to assign each individual to a cluster.

Genetic relationship among the 493 lablab accessions was determined by the unweighted pair group method with arithmetic mean (UPGMA) clustering analysis and principal coordinate analysis (PCoA). Genetic distances [28] between all pairs of the 493 accessions were calculated from the nuclear SSR allele data using the PowerMarker 3.25, and subsequently subjected to UPGMA analysis and neighbor-joining (NJ) analysis using software MEGA 6.0 [42], and PCoA using GenAlEx6.502 software [43]. UPGMA analysis and NJ analysis were conducted with 1000 bootstraps.

Allele data generated from chloroplast SSR markers were used to assign each accession to a haplotype. Then, relationship among haplotypes was analyzed with a median-joining network method [44] using NETWORK software (www.fluxus-engineering.com (accessed on 11 January 2021)).

### 4.5. Development of Lablab Core Collection

A core collection of lablab germplasm was developed by subjecting SSR allele data of all 493 accessions to PowerCore software [45] which apply the advanced M strategy with a heuristic search for establishing core set. Diversity of the core collection was determined by the same software. 

## Figures and Tables

**Table 1:** Variation in 14 morphological traits in 493 lablab accessions.

Attribute	Cultivated		Wild		*t*-Test (Cultivated vs. Wild)
	*uncinatus*	*purpureus + bengalensis*	*uncinatus*	*nomen nominandum*	
**Stem**					
Stem color	Purple, Green	Purple, Green	Purple, Green	Purple, Green	-
**Leave**					
Leave color	Green	Purple, Green	Green	Green	-
**Flower**					
Flower color	Purple	Purple, White	Purple	Purple	-
Day to 1st flower (days)	60–82, average 73.20	17–154, average 93.97	58–98, average 68.57	62–149, average 115.40	ns
**Pod**					
Fresh pod length (cm)	3.20–4.90, average 4.11	3.30–12.50, average 5.98	2.74–3.10, average 2.95	3.00–5.20, average 3.74	**
Fresh pod width (cm)	2.20–2.52, average 2.37	0.63–3.30, average 1.99	1.32–1.78, average 1.56	0.30–1.74, average 1.21	**
Dry pod length (cm)	4.12–5.68, average 4.67	3.10–15.04, average 5.95	3.08–3.46, average 3.25	3.18–4.35, average 3.70	**
Dry pod width (cm)	2.14–2.54, average 2.36	0.86–6.40, average 1.90	1.30–1.88, average 1.65	1.20–1.50, average 1.32	**
Fresh pod color	Green	Purple, Green	Green	Green	
Dry pod color	Brown	Brown	Brown	Brown	
**Seed**					
Seed length (mm)	13.62–14.16, average 13.89	6.08–14.29, average 10.91	7.74–8.92, average 8.31	5.40–7.39, average 6.52	**
Seed width (mm)	10.13–10.30, average 10.21	4.42–10.23, average 7.74	5.60–6.66, average 6.17	4.26–6.13, average 5.00	**
Seed thickness (mm)	6.44–6.72, average 6.58	1.92–8.19, average 4.90	2.06–3.54, average 2.97	2.45–6.65, average 3.45	**
Number of seeds per pod (count)	1.50–2.20, average 1.90	2.20–6.00, average 3.75	2.00–2.20, average 2.09	2.60–4.60, average 3.71	**

ns = non-significant difference, and ** = significant difference at probability level of 0.01.

**Table 2:** Number of alleles per locus (*N*_A_), allele size range, major allele frequency, and polymorphic information content (PIC) of 15 nuclear polymorphic SSR markers in 493 lablab accessions.

Marker Name	*N*_A_	Allele Size Range (Base Pairs)	Major Allele Frequency	Gene Diversity (*H_E_*)	Observed Heterozygosity (*H*_O_)	PIC
c13319_g1_i1	7	184-218	0.9440	0.1078	0.0474	0.1058
c13353_g1_i1	10	252-270	0.7550	0.4055	0.0177	0.3775
c17963_g1_i1	17	200-232	0.5195	0.6844	0.0453	0.6587
c21512_g1_i1	9	225-281	0.7616	0.3903	0.0486	0.3558
c22788_g1_i1	11	333-389	0.8443	0.2809	0.0773	0.2721
c23309_g1_i1	8	273-301	0.7031	0.4765	0.0271	0.4473
Hbp_006	4	170-200	0.9400	0.1137	0.0105	0.1089
Hbp_009	10	374-390	0.7458	0.4175	0.0935	0.3882
Hbp_010	6	240-296	0.9633	0.0715	0.0000	0.0706
Hbp_012	2	256-260	0.9958	0.0084	0.0000	0.0083
KTD184	5	176-187	0.8650	0.2451	0.0082	0.2352
KTD225	10	133-162	0.5514	0.5375	0.0535	0.4410
KTD241	8	144-158	0.6301	0.4995	0.0369	0.4172
KTD245	19	220-310	0.6667	0.5275	0.0380	0.5021
KTD249	5	248-260	0.7589	0.3951	0.0418	0.3615
**Overall**	**131**					
**Mean**	**8.73**		**0.7763**	**0.3441**	**0.0364**	**0.3167**

**Table 3:** Number of alleles per locus (N_A_), major allele frequency (MAF), gene diversity (*H*_E_) and observed heterozygozity (*H*_O_) detected in 493 lablab accessions using 15 nuclear SSR markers.

Type/Region	Subregion	Sample Size	*N*_A_	MAF	*H*_E_	*H*_O_
**Cultivated**		**474**	**112**	**0.80**	**0.3139**	**0.0325**
Africa		120	79	0.78	0.3393	0.0421
	Central	23	23	0.90	0.1320	0.0381
	East	61	61	0.77	0.3565	0.0391
	North	19	19	0.88	0.1250	0.0333
	South	52	52	0.78	0.3158	0.0519
	West	40	40	0.80	0.2917	0.0373
America		22	33	0.88	0.1869	0.0219
	North	20	20	0.93	0.1063	0.0167
	South	32	32	0.87	0.2006	0.0229
Asia		78	78	0.79	0.3018	0.0273
	East	31	31	0.84	0.2467	0.0417
	South	73	73	0.79	0.3175	0.0306
	Southeast	45	45	0.83	0.2370	0.0166
	West	19	19	0.89	0.1259	0.0000
Australia		15	29	0.81	0.2426	0.0249
Europe		5	25	0.85	0.2197	0.0433
unknown		75	58	0.82	0.2568	0.0378
**Wild**		**19**	**79**	**0.52**	**0.6059**	**0.1417**
Africa		17	73	0.52	0.6024	0.1491
	Central	20	1.33	0.82	-	-
	East	38	2.53	0.63	0.4570	0.1044
	South	57	3.80	0.58	0.5489	0.1807
Australia		1	14	0.83	-	-
unknown		1	13	0.77	-	-

**Table 4:** Average number of alleles per locus, major allele frequency (MAF), gene diversity (H_E_) and observed heterozygozity (H_O_) in different subspecies/types of 493 lablab accessions detected by 15 nuclear SSR markers.

Type/Origin	Sample Size	Major Allele Frequency	Average Alleles per Locus	Gene Diversity (*H*_E_)	Observed Heterozygosity (*H*_O_)
**Cultivated**	**474**	**0.80**	**7.47**	**0.3139**	**0.0325**
ssp. *purpureus*	397	0.81	6.60	0.2971	0.0332
ssp. *bengalensis*	33	0.81	2.53	0.2584	0.0306
ssp. *uncinatus*	5	0.91	1.47	0.1222	0.0689
Unknown	39	0.79	3.67	0.3066	0.0251
**Wild**	**19**	**0.52**	**5.27**	**0.6059**	**0.1417**
ssp. *uncinatus*	7	0.80	2.00	0.2775	0.1616
ssp. *nomen nominandum*	12	0.57	4.60	0.5643	0.1338

**Table 5:** Number of alleles, gene diversity, and haplotypes detected in 493 lablab accessions using 6 chloroplast SSR markers.

	VgcpSSR04 (Base Pair)	VgcpSSR05 (Base Pair)	VgcpSSR10 (Base Pair)	VgcpSSR11 (Base Pair)	VgcpSSR12 (Base Pair)	VgcpSSR14 (Base Pair)	
***Haplogroup I***							***Frequency***
**A**	216	206	183	204	236	229	420
***Haplogroup II***							
**B**	222	211	187	206	244	229	5
**C**	222	211	187	206	240	229	1
**D**	222	211	186	212	244	229	5
**E**	222	212	187	202	244	225	1
**F**	222	215	186	202	244	225	1
**G**	222	216	186	202	244	225	1
**H**	222	211	187	202	249	238	2
**I**	222	215	185	206	244	229	3
**J**	224	211	187	202	249	238	1
							***Mean***
**No. alleles per locus**	3	5	4	5	4	4	4.1667
**Gene diversity**	0.1067	0.1105	0.1089	0.1028	0.1027	0.0371	0.0948
**PIC**	0.1015	0.1073	0.1068	0.1011	0.0995	0.0368	0.0922

**Table 6:** Number of alleles per locus, observed heterozygosity, allelic richness, and gene diversity of core collection (47 accessions) of lablab.

Marker	No. of Alleles per Locus	Allelic Richness	Observed Heterozygosity (*H*_O_)	Gene Diversity (*H*_E_)
c13319	7	8	0.1364	0.4556
c22788	11	13	0.1538	0.5562
KTD225	10	12	0.0833	0.727
c17963	17	20	0.0889	0.8054
Hbp006	4	5	0.0455	0.4708
Hbp009	10	13	0.2286	0.68
KTD184	5	7	0.0417	0.5684
KTD249	5	7	0.1277	0.5593
KTD241	8	9	0.0208	0.7029
KTD245	19	21	0.1087	0.8003
c23309	8	8	0.0000	0.6686
Hbp012	2	2	0.0000	0.0416
Hbp010	6	6	0.0000	0.3894
c13353	10	13	0.0667	0.5899
c21512	9	11	0.1163	0.6003
**Overall**	**131**	**10.33**	**0.0812**	**0.5744**

**Table 7:** Details of 14 morphological traits evaluated in the 493 lablab accessions.

Organ	Traits	Evaluation
Stem	Stem color	Green or Purple
Leave	Leave color	Green or Purple
Flower	Flower color	White or Purple
	Day to 1st flower	Number of days from planting to 1st flowering
Pod	Fresh pod length (cm)	Length of straight pod (use 5 pods)
	Fresh pod width (cm)	Maximum width (use 5 pods)
	Dry pod length (cm)	Length of straight pod (use 5 pods)
	Dry pod width (cm)	Maximum width (use 5 pods)
	Fresh pod color	Green or Purple
	Dry pod color	Black or Brown
Seed	Seed length (mm)	Maximum distance from top to bottom of the seed (use 5 seeds)
	Seed width (mm)	Maximum distance from hilum to its opposite side (use 5 seeds)
	Seed thickness (mm)	Maximum distance between both sides of hilum (use 5 seeds)
	Number of seeds per pod (count)	Number of seed per pod

## Data Availability

The data that support the findings of this study are available from the corresponding author upon reasonable request.

## Supplementary Materials

The following supporting information can be downloaded at: https://www.mdpi.com/article/10.3390/plants12010057/s1, Table S1. A list of 493 lablab accessions used in this study. Details of morphological traits, cluster (UPGMA and STRUCTURE) membership, and haplotype group of the 493 accessions are also provided.; Table S2. A list of nuclear and chloroplast SSR markers used in this study; Figure S1. Neighbor-joining tree of 493 lablab accessions based on *D*_A_ genetic distances. The distance was calculated from 15 nuclear SSR markers. (A) The accessions are presented based on their geographical origins. (B) The accessions are presented based on taxonomical classification.
