# Supplementary material for: Molecular Analysis of Genetic Diversity and Structure of the Lablab (Lablab purpureus (L.) Sweet) Gene Pool Reveals Two Independent Routes of Domestication
Source: Plants (Basel). 2022 Dec 22;12(1):57. doi: 10.3390/plants12010057 (PMC9824144; doi:10.3390/plants12010057)
Supplement: Supplementary file 1 [file plants-12-00057-s001.zip › Figure S1.pdf]

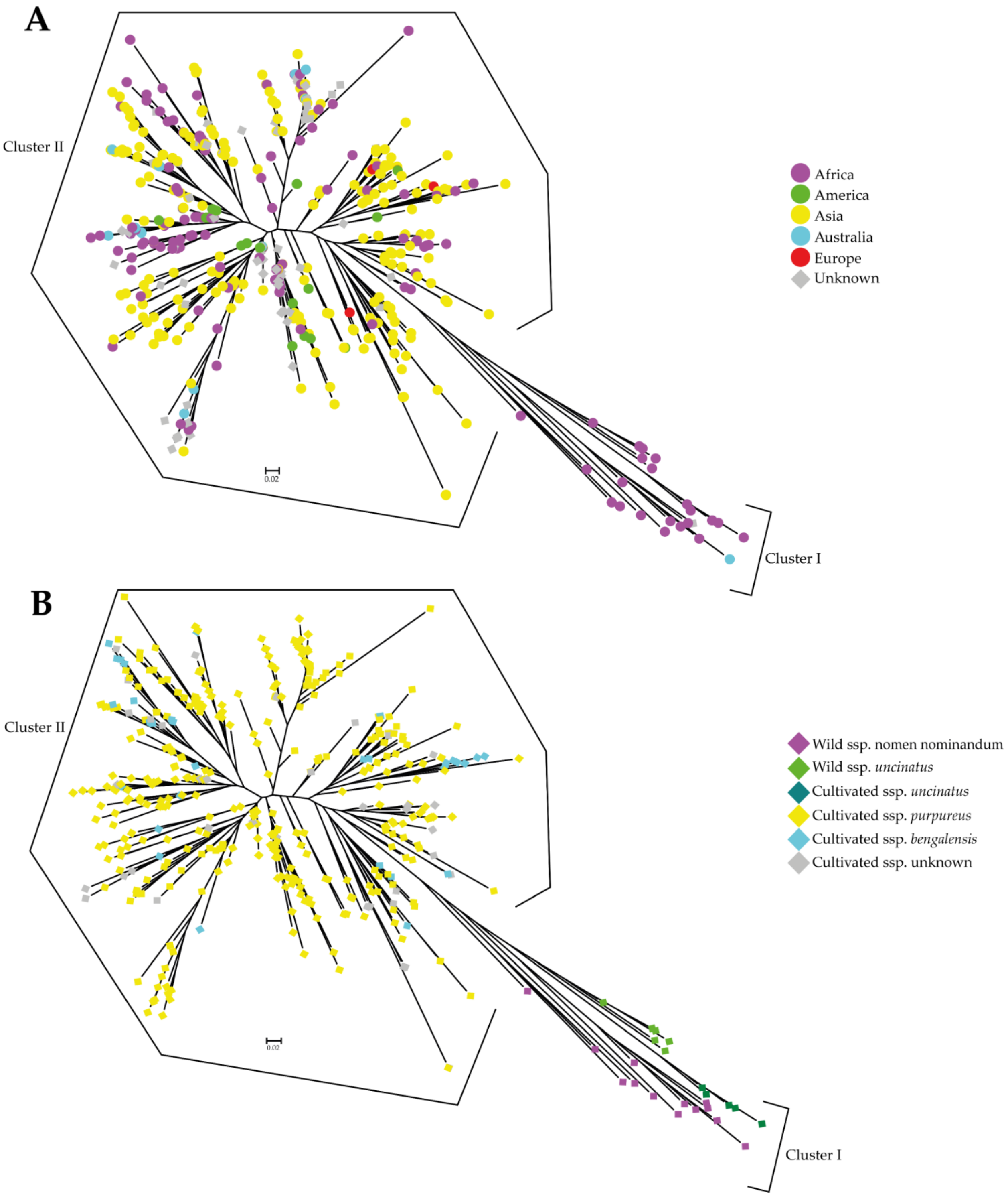

Figure S1. Neighbor-joining tree of 493 lablab accessions based on D_A genetic distances [40]. The distance was calculated from 15 nuclear SSR markers. (A) The accessions are presented based on their geographical origins. (B) The accessions are presented based on taxonomical classification.
